# *Dendropanax morbifera* Léveille extract facilitates cadmium excretion and prevents oxidative damage in the hippocampus by increasing antioxidant levels in cadmium-exposed rats

**DOI:** 10.1186/1472-6882-14-428

**Published:** 2014-10-31

**Authors:** Woosuk Kim, Dae Won Kim, Dae Young Yoo, Hyo Young Jung, Sung Min Nam, Jong Whi Kim, Soon-Min Hong, Dong-Woo Kim, Jung Hoon Choi, Seung Myung Moon, Yeo Sung Yoon, In Koo Hwang

**Affiliations:** Department of Anatomy and Cell Biology, and Research Institute for Veterinary Science, College of Veterinary Medicine, Seoul National University, Seoul, 151-742 South Korea; Department of Biochemistry and Molecular Biology, Research Institute of Oral Sciences, College of Dentistry, Kangneung-Wonju National University, Gangneung, 210-702 Korea; Central Research Center, Egreen Co. Ltd, Seongnam, 463-862 South Korea; Department of Anatomy, College of Veterinary Medicine, Kangwon National University, Chuncheon, 200-701 South Korea; Department of Neurosurgery, Dongtan Sacred Heart Hospital, College of Medicine, Hallym University, Hwaseong, 445-170 South Korea

**Keywords:** *Dendropanax morbifera* extract, Cadmium, Hippocampus, Oxidative stress, Antioxidants

## Abstract

**Background:**

*Dendropanax morbifera* Léveille is used in herbal medicine as a cancer treatment. In this study, we investigated the effects of *Dendropanax morbifera* stem extract (DMS) on cadmium (Cd) excretion from the blood and kidney and brain tissues of rats exposed to cadmium, as well as the effects of DMS on oxidative stress and antioxidant levels in the hippocampus after Cd exposure.

**Methods:**

Seven-week-old Sprague-Dawley rats were exposed to 2 mg/kg of cadmium by intragastric gavage and were orally administered 100 mg/kg of DMS for 4 weeks. Animals were sacrificed and Cd determination was performed using inductively coupled plasma mass spectrometry. In addition, the effects of Cd and/or DMS on oxidative stress were assayed by measuring reactive oxygen species production, protein carbonyl modification, lipid peroxidation levels, and antioxidant levels in hippocampal homogenates.

**Results:**

Exposure to Cd significantly increased Cd content in the blood, kidneys, and hippocampi. DMS treatment significantly reduced Cd content in the blood and kidneys, but not in the hippocampi. Exposure to Cd significantly increased reactive oxygen species production, protein carbonyl modification, lipid peroxidation, total sulfhydryl content, reduced glutathione content, and glutathione reductase activity. In contrast, Cu, Zn-superoxide dismutase (SOD1), catalase (CAT), glutathione peroxidase (GPx), and glutathione-*S*-transferase (GST) activity in the hippocampus were significantly decreased after exposure to Cd, and administration of DMS significantly inhibited these Cd-induced changes.

**Conclusion:**

These results indicate that DMS facilitates cadmium excretion from the kidneys, reduces cadmium-induced oxidative stress in the hippocampus, and modulates SOD1, CAT, GPx, and glutathione-*S*-transferase activities.

## Background

*Dendropanax morbifera* Léveille is mainly distributed in southwestern Korea. This tree was initially used for the production of golden varnish [1], but the leaves, stems, roots, and seeds contain compounds that are used in traditional medicine for the treatment of skin and infectious diseases, headaches, and other maladies [2]. The polyacetylene compounds in *Dendropanax morbifera* leaves have anti-complement, anti-diabetic, and anti-atherogenic activities [1, 3–5].

Cadmium (Cd) is one of the most harmful heavy metals in nature and it is released into the environment through mining and smelting. The main sources of Cd exposure include production of certain types of batteries, intake of contaminated food and water, and inhalation of tobacco smoke or polluted air [6, 7]. Cd exposure generates free radicals such as superoxide radicals, hydroxyl radicals, and nitric oxide [8]. Free radical accumulation in animals is harmful to tissue types in many organs, including the brain and spinal cord. The nervous system is highly susceptible to damage induced by free radicals because nervous tissue contains high levels of unsaturated fatty acids and iron [9]. Cd exposure induces oxidative DNA damage due to excess oxygen-derived free radicals [10, 11] and depletes antioxidant levels, resulting in a state of oxidant/antioxidant imbalance. Therefore, modulation of antioxidant levels in the brain may be an effective strategy with which to reduce Cd-induced neurotoxicity.

Several approaches have been used to promote or facilitate the excretion of Cd from the body because it has a long biological half-life (approximately 30 years in humans) and readily accumulates in tissues [12]. Debarked *Dendropanax morbifera* stem extract (DMS) decreases 2,2-diphenyl-1-picrylhydrazyl radical levels, and its antioxidant capacity is greater than that of other organ extracts from *D. morbifera* [13]. In addition, rutin, a bioflavonoid isolated from *D. morbifera,* significantly decreases rotenone-induced generation of reactive oxygen species in SH-SY5Y cells and mitochondrial membrane potential depletion [14]. However, there are no reports on the effects of DMS on oxidative stress and antioxidant enzyme activity after Cd exposure. Therefore, in this study, we investigated the effects of DMS treatment on Cd excretion from the kidney and hippocampus, and on antioxidant enzyme activity and lipid peroxidation in the hippocampus of Cd-exposed rats.

## Methods

### Experimental animals

Male Sprague-Dawley rats were purchased from Orient Bio Inc. (Seongnam, South Korea). Rats were housed in a conventional animal facility at 23°C with 60% humidity, a 12 h/12 h light/dark cycle, and free access to food and tap water. The handling and care of the animals conformed to the guidelines established in order to comply with current international laws and policies (NIH Guide for the Care and Use of Laboratory Animals, NIH Publication No. 85-23, 1985, revised 1996), and were approved by the Institutional Animal Care and Use Committee (IACUC) of Seoul National University (SNU-130522-1). All of the experiments were conducted with an effort to minimize the number of animals used and the suffering caused by the procedures used in the study.

### Preparation of DMS

Fresh *Dendropanax morbifera* Léveille was purchased from a local market on Jeju Island in Korea. The plant was authenticated by two practitioners of traditional Asian medicine, and a voucher specimen was deposited with Egreen Co. Ltd. (deposition number: 2013-001). Stems from the plant samples (100 g) were chopped, blended, soaked in 2 L of 80% ethanol, and refluxed three times at 20°C for 2 h. The insoluble materials were removed by centrifugation at 10,000 × *g* for 30 min, and the resulting supernatant was concentrated and freeze-dried to yield a powder (yield: 4.7%). Before each experiment, the dried extract was dissolved in distilled and deionized water.

### Administration of Cd and DMS

Cadmium chloride was obtained from Sigma-Aldrich (St. Louis, MO, USA). Animals were divided into 4 treatment groups (*n* = 29 in each group): a control group, a group treated with 100 mg/kg DMS, a group treated with 2 mg/kg Cd, and a group treated with Cd and DMS. Cd and/or DMS were administered orally to 7-week-old rats once a day for 4 weeks.

### Cd level in the blood, brain, and kidney

To measure Cd concentration in the blood and brain and kidney tissues, control, DMS-, Cd-, and Cd-DMS-treated rats (*n* = 7 in each group) were anesthetized with 100 mg/kg of Zoletil 50^®^ (Virbac, Carros, France) and the blood, hippocampi, and kidneys were extracted. Blood samples were allowed to clot, after which they were centrifuged for 30 min at 1,000 *g* to separate the serum. Hippocampi and kidneys were weighed in glass vessels, and tissues were digested by adding 3-8 mL of HNO_3_ at 130°C for 3 h, after which 2 mL of H_2_O_2_ was added and the samples were heated for 1 h. Serum and digested samples were transferred to polypropylene flasks for Cd determination. Cd determination was performed by using inductively coupled plasma mass spectrometry (ICP-MS; PerkinElmer Sciex, Thornhill, Canada).

### Measurement of reactive oxygen species (ROS) production in the hippocampus

The effects of Cd and/or DMS on ROS production in the hippocampus of control, DMS-, Cd-, and Cd-DMS-treated rats (*n* = 5 in each group) were assessed using the fluorescent probe 2′,7′-dichlorofluorescin diacetate (DCFH-DA) [15]. Intracellular ROS oxidize DCFH-DA to dichlorofluorescein (DCF), an intense fluorescent chemical. Four weeks after Cd treatment, rats in each group were deeply anesthetized and euthanized by decapitation. Brain mitochondria were obtained as described previously [16]. Mitochondrial protein quantification was determined by the Bradford method [17] using bovine serum albumin (BSA) as the standard. Mitochondria isolated from different groups (0.5 mg/mL) were incubated with 10 μM DCFH-DA at 37°C for 60 min, and the fluorescence intensity of DCF was measured at an excitation wavelength of 488 nm and emission wavelength of 527 nm in a microplate reader (SpectraMax M5, Molecular Devices LLC, Sunnyvale, CA, USA).

### Measurement of lipid peroxidation in hippocampal homogenates

The effects of Cd and/or DMS on lipid peroxidation levels in control, DMS-, Cd-, and Cd-DMS-treated rats (*n* = 5 in each group) were assessed by measuring malondialdehyde (MDA) formation using the Bioxytech MDA-586 kit (Oxis Research, Portland, OR, USA). Briefly, bilateral hippocampi were collected using a surgical blade and the left part was homogenized in 20 mM PBS (pH 7.4) containing 5 mM butylated hydroxytoluene. After centrifugation of the homogenates at 3000 × *g* for 10 min at 4°C, the supernatants were collected. For each reaction, 10 μL of probucol and 640 μL of diluted R1 reagent (1:3 of methanol:*N*-methyl-2-phenylindole) were added and mixed with 150 μL of 12 N HCl. Each reaction was incubated at 45°C for 60 min and centrifuged at 10,000 × *g* for 10 min. The supernatant was collected and MDA formation was determined by measuring the absorbance at 586 nm. MDA data were normalized to the protein concentration of each sample.

### Protein carbonyl levels

To elucidate the effects of Cd and/or DMS on protein modification, carbonylation of proteins as a result of oxidative stress was determined in control, DMS-, Cd-, and Cd-DMS-treated rats (*n* = 5 in each group) by the Levine method [18]. The color intensity of the supernatant was measured at 370 nm using a spectrophotometer against 2 M HCl. Carbonyl content was calculated using the molar extinction coefficient (21 × 10^3^ L/mol cm), and results were expressed as nmol/mg of protein.

### Measurement of antioxidant activity in hippocampal homogenates

To elucidate the effects of Cd and/or DMS on Cu, Zn-superoxide dismutase 1 (SOD1), catalase (CAT), and glutathione peroxidase (GPx), the activity of these enzymes was measured in control, DMS-, Cd-, and Cd-DMS-treated rats (*n* = 7 in each group). Briefly, the right part of the hippocampus (matching the right part used for measurement of lipid peroxidation) was homogenized in 10 mM Tris buffer containing 1 mM EDTA and 1 mM PMSF. The homogenates were centrifuged at 600 × *g* for 10 min, and then centrifuged at 13,000 × *g* for 20 min at 4°C.

SOD1 activity was measured by monitoring its capacity to inhibit the reduction of ferricytochrome *c* by xanthine/xanthine oxidase as described by McCord and Fridovich [19]. SOD1 activity staining was performed by electrophoresis in 10% native polyacrylamide gels and visualized as described by Beauchamp and Fridovich [20]. Briefly, the gel was soaked in 2.45 mM nitroblue tetrazolium solution for 15 min, followed by 30 min in 28 mM *N*, *N*, *N′′*, *N′′*-tetramethylethylene diamine and 28 μM riboflavin in 0.36 mM potassium phosphate buffer (pH 7.8). The gel was exposed to a fluorescent light source until the bands showed maximum resolution.

CAT activity was assayed at 25°C by determining the rate of H_2_O_2_ degradation in 10 mM of potassium phosphate buffer (pH 7.0) according to Aebi’s method [21]. An extinction coefficient of 43.6 mM/cm was used for the calculations. One unit is defined as 1 pmol of H_2_O_2_ consumed per min and the specific activity is reported as units per mg of protein.

GPx activity was assayed by measuring nicotinamide adenine dinucleotide phosphate (NADPH) oxidation with *t*-butyl-hydroperoxide as a substrate according to the method of Maral et al. [22]. Briefly, the reaction was carried out at 25°C in 600 μL of a solution containing 100 mM potassium phosphate buffer (pH 7.7), 1 mM EDTA, 0.4 mM sodium azide, 2 mM glutathione, 0.1 mM NADPH, 0.62 U of glutathione reductase, and 50 μL of homogenate.

### Measurement of glutathione-related enzymes in hippocampal homogenates

The effects of Cd and/or DMS on glutathione-related enzymes in the hippocampus of control, DMS-, Cd-, and Cd-DMS-treated rats (*n* = 5 in each group) were investigated using the tissue samples obtained for the measurement of protein carbonyl levels. Glutathione-*S*-transferase (GST) activity was determined spectrophotometrically using 1-chloro-2,4-dinitrobenzene as a substrate [23]. Glutathione reductase (GR), which has been shown to utilize NADPH to convert oxidized glutathione (GSSG) to the reduced form (GSH), was assayed by the method of Horn and Burns [24].

### Statistical analysis

All data are expressed as mean ± standard error of the mean (SEM). To determine the effects of Cd and DMS, differences between the means were statistically analyzed using two-way analysis of variance (ANOVA) with repeated measures and Bonferroni’s post hoc test.

## Results

### Quantification of Cd content in kidney and brain tissues

The ICP-MS method was used to determine Cd concentrations in serum, kidney, and hippocampal tissues. Cd concentrations in serum, kidney, and hippocampal tissues were similar in the control and DMS-treated groups. In the Cd-treated group, Cd concentrations in the serum and kidney were significantly higher than those in the control group. In contrast, there was no significant difference in the Cd concentration in the hippocampus between the control and DMS-treated groups, although the level was increased 1.57-fold in the control group. In the Cd-DMS-treated group, Cd concentrations in the serum and kidneys were significantly lower than those in the Cd-treated group (Table 1).

**Table 1:** **Effects of cadmium (Cd) and/or** ***Dendropanax morbifera*** **stem extract (DMS) on Cd concentration (μg/g) in the blood and hippocampal and kidney tissues of rats**

Tissue	Control	DMS	Cd	Cd/DMS
Blood	4.12 ± 1.12	3.48 ± 0.84	39.7 ± 4.12*	26.9 ± 5.36*^,#^
Hippocampus	0.014 ± 0.0009	0.012 ± 0.0012	0.022 ± 0.0012	0.019 ± 0.0011
Kidney	0.015 ± 0.0028	0.014 ± 0.0041	4.365 ± 1.4105*	3.523 ± 1.1254*^,#^

* Indicates a significant difference between the control and Cd groups (*P* <0.05); ^*#*^ Indicates a significant difference between the Cd and Cd/DMS groups (*P* <0.05; *n* = 7 per group). The data represent the mean ± standard error of the mean (SEM).

### Effects of Cd and/or DMS on ROS formation in hippocampal homogenates

**Figure 1:**
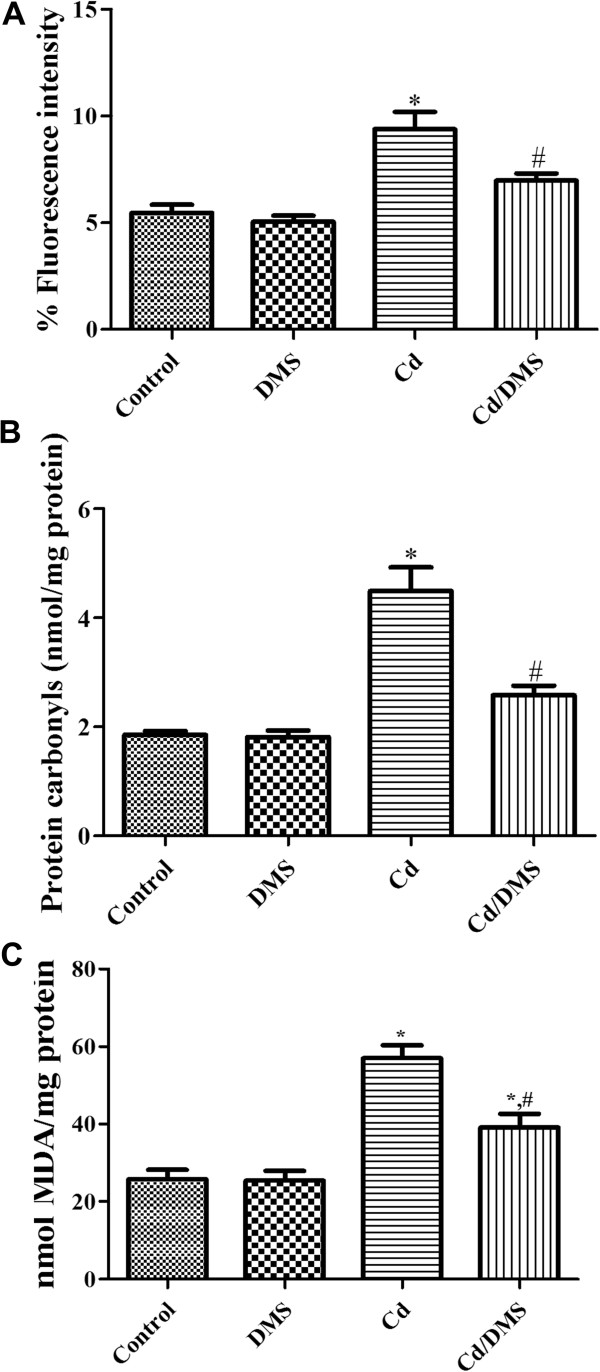
**Levels of intracellular reactive oxygen species production as determined by 2′,7′-dichlorofluorescein (DCF) levels (A), protein carbonyl levels (B), and malondialdehyde (MDA) levels (C) in the hippocampi of control, DMS-, Cd-, and Cd-DMS-treated rats.** * Indicates a significant difference between the control and Cd groups (*P* <0.05); ^*#*^ indicates a significant difference between the Cd and Cd/DMS groups (*P* <0.05; *n* = 5-7 per group). DCF, protein carbonyl, and MDA levels are significantly higher in the Cd group and the administration of DMS to Cd-exposed rats leads to a decrease in DCF, protein carbonyl, and MDA levels. The data represent means ± standard error of the mean (SEM).

DCF fluorescence levels were similar in the control and DMS-treated groups. DCF fluorescence was significantly increased (1.71-fold) in the Cd-treated group in comparison with that of the control group. However, the MDA level of the Cd-DMS-treated group was significantly decreased to 74.4% of that of the Cd-treated group (Figure 1A).

### Effects of Cd and/or DMS on protein carbonyl levels in hippocampal homogenates

Protein carbonyl levels were similar in the control and DMS-treated groups. However, the protein carbonyl level of the Cd-treated group was significantly increased (2.4-fold) in comparison with that of the control group. The protein carbonyl level of the Cd-DMS-treated group was significantly decreased to 57.4% of that of the Cd-treated group (Figure 1B).

### Effects of Cd and/or DMS on lipid peroxidation in hippocampal homogenates

MDA levels in hippocampal homogenates were 25.8 and 25.4 nmol/mg protein in the control and DMS-treated groups, respectively. The MDA level of the Cd-treated group was 2.2-fold higher than that of the control group. However, the MDA level of the Cd-DMS-treated group was significantly lower (39.1 nmol/mg protein) than that of the Cd-treated group (Figure 1C).

### Effects of Cd and/or DMS on SOD1, CAT, and GPx activities in hippocampal homogenates

**Figure 2:**
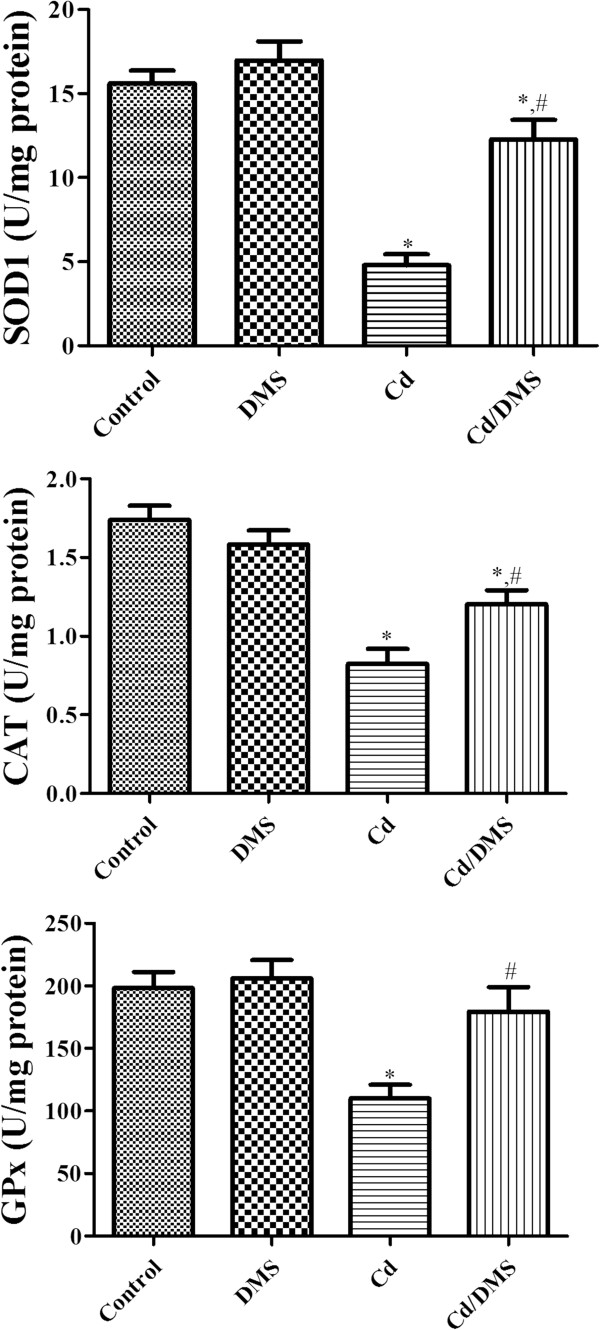
**Cu, Zn-Superoxide dismutase 1 (SOD1), catalase (CAT), and glutathione peroxidase (GPx) activity in the hippocampi of control, DMS-, Cd-, Cd-DMS-treated rats.** * Indicates a significant difference between the control and Cd groups (*P* <0.05); ^*#*^ indicates a significant difference between the Cd and Cd/DMS groups (*P* <0.05; *n* = 7 per group). Antioxidant enzyme activities are significantly lower in the Cd group and the administration of DMS to Cd-exposed rats ameliorates the reduction of enzyme activities. The effect on GPx activity is greater than that on SOD1 or CAT activities. The data represent means ± standard error of the mean (SEM).

SOD1, CAT, and GPx activities in the control group were 15.61, 1.739, and 198.1 U/mg protein, respectively. In the DMS-treated group, SOD1 and GPx activities were slightly higher than those in the control group. However, CAT activity was lower than that of the control group. In the Cd-treated group, SOD1, CAT, and GPx activities were significantly lower than those in the control group. The effect of Cd exposure on SOD1 activity was its most pronounced effect. SOD1, CAT, and GPx activities in the Cd-DMS-treated group were significantly higher than those in the Cd-treated group, and the greatest increase was in GPx activity (Figure 2).

### Effects of Cd and/or DMS on glutathione-related enzymes in hippocampal homogenates

**Figure 3:**
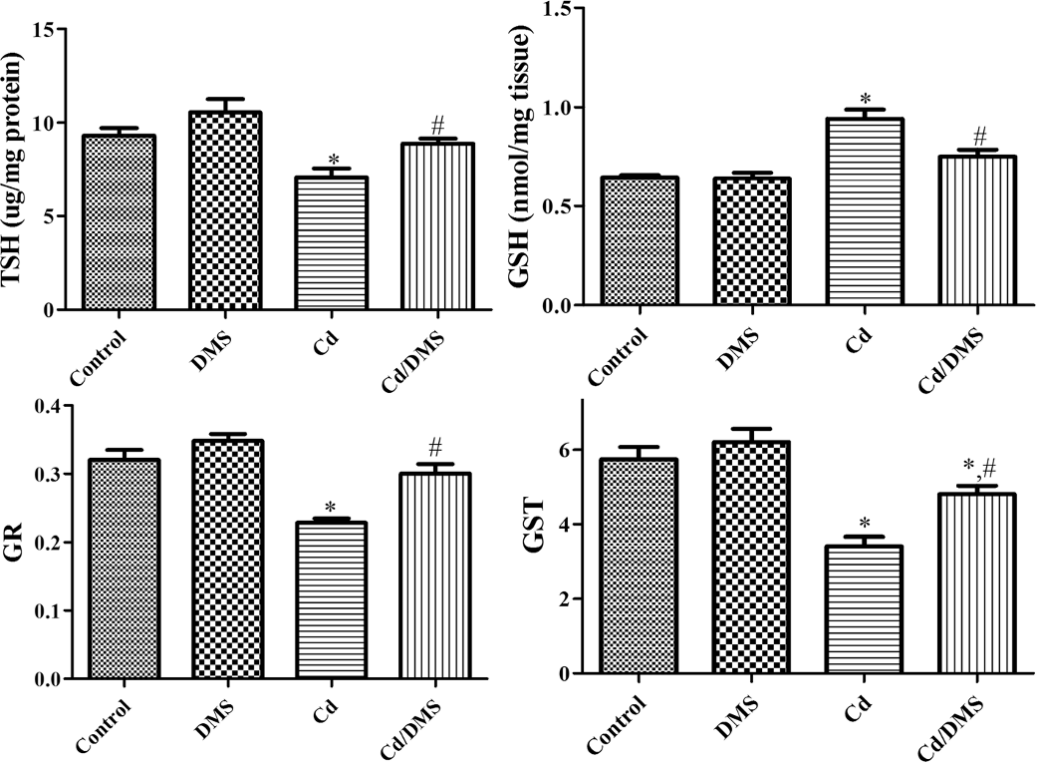
**Total sulfhydryl groups (TSH), reduced glutathione (GSH), glutathione reductase (GR), and glutathione-** ***S*** **-transferase (GST) in the hippocampi of control, DMS-, Cd-, Cd/DMS-treated rats.** * Indicates a significant difference between the control and Cd groups (*P* <0.05); ^*#*^ indicates a significant difference between the Cd and Cd/DMS groups (*P* <0.05; *n* = 5 per group). GR activity, TSH levels, and GSH levels are significantly increased after exposure to Cd, while GST activity is significantly increased. The administration of DMS to Cd-exposed rats ameliorates or reverses the changes of enzyme activities or levels. The data represent means ± standard error of the mean (SEM).

In the control and DMS-treated groups, GSH level, TSH (total sulfhydryl groups) level, and the activities of GR and GST were similar in the hippocampal homogenates. In the Cd-treated group, TSH level and GR activity were significantly decreased to 53.5% and 37.8% of those of the control group, respectively. In contrast, GST activity in the Cd-treated group was significantly increased to 147.7% of that of the control group. In the Cd-DMS-treated group, TSH level and GR activity were significantly increased to 172.9% and 174.7% of those of the control group, respectively. GST activity in the Cd-DMS-treated group was significantly decreased to 58.5% of that of the Cd-treated group (Figure 3).

## Discussion

Cd causes toxicity by disturbing the cellular homeostasis of essential metal ions such as copper and zinc. In addition, Cd affects antioxidant enzymes, which use copper and zinc, through oxidation or peroxidation in the brain [25]. In contrast, DMS has high ferric-reducing ability and contains high levels of phenolic compounds [13]. Therefore, we investigated the effects of DMS on Cd-induced oxidative stress in the hippocampus. Chronic administration of Cd significantly increased Cd levels in kidney and hippocampal tissues. Supplementation with DMS markedly decreased the Cd concentration in the kidneys, but Cd levels were only slightly decreased in hippocampal tissue.

Cd can affect the integrity and permeability of the vascular endothelium, penetrate the blood-brain barrier, and accumulate in the brain [26–28]. In this study, we measured mitochondrial ROS formation after Cd exposure based on the conversion of DCFH-DA to DCF. Exposure to Cd significantly increased ROS formation. In addition, we observed decreased TSH content and increased protein carbonylation because the oxidative effect of Cd is associated mainly with the depletion of sulfhydryl group-containing compounds [29]. Cd administration significantly decreased TSH content and increased protein carbonylation in the hippocampus. This result confirmed those of previous studies, which showed that Cd administration significantly increased ROS-mediated reactions in the brain, including the hippocampus [30], and kidney [31]. We also estimated lipid peroxidation by measuring MDA level, which is a major oxidation product of peroxidized polyunsaturated fatty acids [32]. Repeated administration of Cd significantly increased MDA levels in the hippocampus. This result is consistent with previous studies showing that exposure to Cd for 30 days significantly increases MDA levels in hippocampal tissue [33, 34]. However, supplementation with DMS significantly reduced MDA levels in the hippocampus. This suggests that DMS potently inhibits Cd-induced lipid peroxidation in the hippocampus. This result is in accordance with a recent study showing that rutin isolated from *Dendropanax morbifera* Léveille significantly decreases rotenone-induced generation of reactive oxygen species in SH-SY5Y cells [14].

In this study, we also determined the effects of DMS and/or Cd on the activity of SOD1, CAT, and GPx in the hippocampus because Cd has been shown to inhibit antioxidant enzymes [33–38]. We found that Cd exposure for 4 weeks significantly reduced the activities of SOD1, CAT, and GPx in the hippocampus. These data are in agreement with those of previous studies showing that GPx, SOD1, Mn-SOD, and CAT activities in the hippocampus are significantly decreased after Cd exposure for 30 days [33, 34]. Cd attacks intracellular sulfhydryl groups and disrupts organelles [39], because most antioxidant enzymes become inactive when Cd binds to their active sites [40]. Supplementation with DMS prevented the Cd-induced inhibition of SOD1, CAT, and GPx activities in the hippocampus, suggesting that the antioxidant effects of DMS may attenuate Cd-induced neurotoxicity in the hippocampus.

We focused on glutathione-related enzymes because the increase in activity after DMS treatment in Cd-exposed rats was most prominent for GPx, among the antioxidative enzymes. Exposure to Cd significantly decreased GR activity, TSH level, and GSH level, while GST activity was significantly increased. The decrease of GR activity, TSH level, and GSH level after Cd exposure could be responsible for increased ROS levels and subsequent lipid peroxidation and protein carbonylation in the brain, because GSH can directly scavenge ROS or act as a substrate for GPx and GST in the detoxification of hydrogen peroxide [41]. DMS administration significantly ameliorated changes in glutathione-related enzymes in the hippocampus caused by Cd exposure, and this effect may be associated with direct reductions in ROS levels.

## Conclusion

The administration of DMS promotes the excretion of Cd from the kidney and ameliorates Cd-induced increases in ROS, lipid peroxidation, and protein carbonyl levels, which are modified by oxidative stress. In addition, DMS efficiently attenuates Cd-induced deficits in SOD1, CAT, GPx, and glutathione-related enzyme activities in the hippocampus.
